# Clinical Characteristics of Fulminant Type 1 Diabetes Compared with Typical Type 1 Diabetes: One-Year Follow-Up Study from the Guangdong T1DM Translational Medicine Study

**DOI:** 10.1155/2020/8726268

**Published:** 2020-02-19

**Authors:** Daizhi Yang, Yongwen Zhou, Sihui Luo, Xueying Zheng, Ping Ling, Liling Qiu, Wen Xu, Hua Liang, Bin Yao, Jianping Weng, Jinhua Yan

**Affiliations:** ^1^Department of Endocrinology and Metabolism, The Third Affiliated Hospital of Sun Yat-sen University, Guangdong Provincial Key Laboratory of Diabetology, Guangzhou 510630, China; ^2^Department of Endocrinology and Metabolism, The First Affiliated Hospital of USTC, Division of Life Sciences of Medicine, University of Science and Technology of China, Hefei 230026, China

## Abstract

**Background:**

Fulminant type l diabetes mellitus (FT1DM) is a subtype of type 1 diabetes mellitus (T1DM) with abrupt onset, but data on its progression was limited. This study was aimed at exploring the clinical features through one-year follow-up.

**Methods and Materials:**

Patients with T1DM finishing at least one-year follow-up from June 2011 to July 2018 were enrolled from Guangdong Type 1 Diabetes Translational Medicine Study. Patients who fulfilled the respective criteria were categorized as an FT1DM group and a typical T1DM group (TT1DM). The 1 : 4 propensity score matching based on onset age, duration, and gender was performed between the FT1DM and TT1DM groups. Characteristics at the onset and after one-year follow-up were compared between the two groups.

**Results:**

A total of 53 patients with FT1DM and 212 matched patients with TT1DM were included. At the onset, there was a shorter duration of symptomatic period before diagnosis observed in the FT1DM group than in the TT1DM group (2 [1, 7] vs. 30 [10, 60] days, *P* < 0.001). FT1DM patients had higher plasma glucose levels and higher percentage of diabetes ketoacidosis (*P* < 0.001, respectively). Both fasting and postprandial C-peptide levels (FCP and PCP, respectively) in FT1DM were significantly lower (*P* < 0.001). At enrollment, the duration of diabetes was 0.03 (0.00, 0.81) and 0.07 (0.00, 1.11) years and the level of HbA1c was 7.21 ± 1.56% and 10.06 ± 3.23% (*P* < 0.001) in the FT1DM and TT1DM groups, respectively. After one year, both FCP and PCP were still significantly lower in the FT1DM group (*P* < 0.001, 0.022) and the HbA1c level was similar between the two groups (*P* = 0.128). The level of HDL-C in FT1DM was significantly higher than that in the TT1DM group at enrollment (*P* = 0.019), and the change from enrollment was significantly greater than that in the FT1DM group (*P* = 0.042).

**Conclusion:**

Patients with FT1DM had more severe metabolic derangement and deficiency of insulin secretion than patients with TT1DM at the onset, but glycaemic and metabolic control was not worse than that in TT1DM.

## 1. Introduction

Fulminant type 1 diabetes (FT1DM), firstly proposed in 2000 by Imagawa, is an independent subtype of idiopathic diabetes which was mostly reported in the Asian population such as Japan, Korea, and China [1–4]. It was characterized by the near-normal glycated haemoglobin (HbA1c) level, rapid onset of hyperglycaemia with ketoacidosis, absolute beta-cell dysfunction, elevated serum levels of pancreatic enzymes, and so on. Multiple factors including genetics, viral infection, and autoimmunity were thought to contribute partly to the mechanism of FT1DM, but the precise pathways remained unknown [5]. In previous studies, FT1DM showed more severe ketoacidosis at the disease onset than autoimmune type 1A diabetes [1]. However, the studies on the clinical progression of FT1DM were sporadic [1, 6, 7]. Due to the lack of endogenous insulin secretion in FT1DM from the onset, strict glycaemic control is usually difficult and glucose levels tend to be erratic, which might be difficult for the daily care and prevention of complications in this population. Therefore, to better understand the diseases, we characterized metabolic control in FT1DM after one-year follow-up via the large-scale database of T1DM in China.

## 2. Materials and Methods

### 2.1. Study Design

The Guangdong Type 1 Diabetes Mellitus translational study (GTT study) was a prospective multicentre hospital-based register study, conducted in 16 tertiary hospitals throughout 12 cities of Guangdong, China. The inclusive criteria and detailed protocols have been reported previously [8, 9]. Newly diagnosed and priorly diagnosed patients with T1DM who visited the participating hospitals consecutively from June 2011 to July 2018 and finished at least one-year follow-up were enrolled in the study.

Based on the diagnostic criteria of FT1DM proposed in 2012 [10], the inclusion criteria of the FT1DM group were as follows: (1) occurrence of diabetic ketosis or ketoacidosis (DK/DKA) soon (approximately 7 days) after the onset of hyperglycaemic symptoms, (2) plasma glucose level ≥ 16 mmol/l (≥288 mg/dl) and HbA1c level < 8.7% at the first visit, and (3) fasting serum C-peptide level < 0.10 nmol/l and <0.17 nmol/l after intravenous glucagon load (or after meal) at the onset. Those who were compatible with the last two criteria but had the duration of the disease before the start of insulin treatment for more than one to two weeks were highly suspected and also assigned into the FT1DM group. Typical type 1 diabetes was defined as follows [8]: (1) obvious symptoms of a diabetes-related metabolic disorder, (2) positive diabetic autoantibodies at any time (glutamic acid decarboxylase antibody (GADA), insulinoma-associated protein 2 (IA-2A), and zinc transporter 8 antibody (ZnT8A)), (3) previous DK/DKA, and (4) fasting and stimulated C-peptide levels < 0.2 nmol/l.

### 2.2. Measurements and Definitions

Data collection was conducted in participating hospitals. Onset information including medical history, accompanied symptoms (pancreatitis, infection, and conscious disorder), clinical characteristics, and biological indicators was collected from the medical reports. And then, data about demographics, anthropometric measurements, and serological tests were collected by trained physicians and nurses at enrollment and then once a year.

HbA1c was measured using affinity chromatography with an Afinion™ AS100 point-of-care device HbA1c analyzer (Axis-Shield Diagnostics Ltd., Dundee, Scotland; reference range 4.3–6.1%, total coefficients of variation < 3%) at enrollment and then once a year in participating hospitals. Serum creatinine (Cr), blood urine nitrogen (BUN), lipid profiles, and C-peptide (fasting and after 2 h mixed meal) were measured centrally at enrollment and after one-year follow-up. Lipid profiles, Cr, and BUN were determined by an enzymatic colorimetric test with a Hitachi 7600 autoanalyzer. Fasting/postprandial C-peptide was measured with an iodine (125I) radioimmunoassay kit (Beijing North Institute of Biological Technology, Beijing, China; intrabatch and interbatch coefficients of variation 0.46 and 0.99%, respectively).

Autoantibodies against the 65 kDa isoform of GADA, IA-2A, and ZnT8A were analyzed centrally at enrollment using fasting serum with a radiobinding assay confirmed by the Islet Autoantibody Standardization Program (assay sensitivity and specificity were 64 and 98% for GADA, 64 and 100% for IA-2A, and 36 and 98% for ZnT8A, respectively) at the First Affiliated Hospital of Nanjing University. Patients with positive results for at least 1 antibody titer tested (GADA titer≧0.042 was seen as positive, ZnT8A titer≧0.054 was seen as positive, and IA‐2A titer≧0.018 was seen as positive) were considered positive for diabetes autoantibodies.

Among the adult patients, the insulin resistance (IR) was calculated according to the following formula [11]: ln GDR = 4.964 − 0.121 × HbA1c (%) − 0.012 × diastolic blood pressure (mmHg) − 1.409 × (waist/hip ratio). Weight and height measurements were used to calculate BMI (kg/m^2^).

### 2.3. Statistical Analysis

The 1 : 4 propensity score matching (PSM) analysis generated with the MatchIt package in the R program (3.6.1) was used to minimize the effect of baseline characteristic imbalances between the FT1DM and TT1DM groups. The logistic model included the following variables: onset age, diabetic duration at enrollment, and gender. A standardized mean difference (SMD) with an absolute value less than 0.10 was taken to indicate a negligible difference in the covariates between groups [12]. The SMD of onset age, diabetic duration, and gender was 0.073, 0.099, and 0.001, respectively. After PSM, data was analyzed by SPSS22.0 software (IBM Corporation, New York, NY, USA). The Kolmogorov-Smirnov test was used to identify Gaussian distribution. Nonparametric data was presented as median and interquartile range while parametric ones were clarified as mean ± standard deviation. Statistical differences between groups were analyzed by the Mann–Whitney *U* tests, *χ*^2^ or Fisher's exact test, or *t*-test where appropriate. Changes from enrollment to one-year follow-up were analyzed between the two groups using analysis of covariance with corresponding values at enrollment adjusted. A two-sided *P* value < 0.05 was considered statistically significant.

## 3. Results

From June 2011 to July 2018, a total of 1583 T1DM patients registered and finished at least one-year follow-up in the Guangdong Type 1 Diabetes Mellitus translational study (GTT study). Among them, 53 patients (male/female, 22/31) were selected into the FT1DM group and 1530 patients were selected into the TT1DM group according to the respective criteria. After nearest-neighbor 1 : 4 PSM, 212 matched patients in the TT1DM group were enrolled into analysis.

### 3.1. Characteristics at the Onset

As is shown in Table 1, the onset age in the FT1DM group and TT1DM group was 31.28 ± 11.94 and 32.47 ± 14.22 years, respectively (*P* = 0.445). The BMI in the FT1DM group was higher than that in the TT1DM group (*P* = 0.015). Among 31 female patients with FT1DM, 8 (25.81%) patients developed diabetes during pregnancy and fetal demise occurred in 7 patients. Among 93 female patients with TT1DM, 4 (4.30%) patients developed diabetes during pregnancy and but none of them resulted in fetal demise.

At the onset, 88.7% of FT1DM patients presented with DKA while there were only 40.1% at the onset with DKA in the TT1DM group. The duration of hyperglycaemic symptoms before diagnosis was shorter in the FT1DM group (2 [1, 7] vs. 30 [10, 60] days, *P* < 0.001). Compared with the TT1DM group, the plasma glucose level at the onset in the FT1DM group was higher (34.09 ± 12.35 vs. 24.98 ± 9.64 mmol/l, *P* < 0.001) while the HbA1c level was significantly lower (6.42 ± 0.72 vs. 12.13 ± 3.40%, *P* < 0.001). Meanwhile, both the fasting (0.01 [0.00, 0.03] vs. 0.09 [0.04, 0.23] nmol/l, *P* < 0.001) and 2 h postprandial C-peptide levels (0.02 [0.01, 0.05] vs. 0.20 [0.09, 0.54] nmol/l, *P* < 0.001) were significantly lower in the FT1DM group.

At the onset, FT1DM patients had significantly lower arterial pH, arterial and blood bicarbonate level, serum sodium level, and serum LDL-C level and higher serum potassium level and *β*-hydroxybutyric acid level. The level of leukocytes and neutrophils was also significantly higher in the FT1DM group. Infectious and conscious symptoms accompanied at the onset were more frequent in the FT1DM group (*P* < 0.001, *P* < 0.001). Other parameters including TC, TG, and serum HDL-C were similar between the two groups.

### 3.2. Characteristics at Enrollment and after One-Year Follow-Up

Changes of the characteristics between enrollment and one-year follow-up of the two groups are shown in Table 2. At enrollment, the duration of diabetes was 0.03 (0.00, 0.81) and 0.07 (0.00, 1.11) years in the FT1DM and TT1DM groups, respectively. HbA1c level in FT1DM was significantly lower than that in the TT1DM group (7.21 ± 1.56 vs. 10.06 ± 3.23%, *P* < 0.001). After one-year follow-up, the level of HbA1c was similar between the two groups (*P* = 0.128). The change in HbA1c level from enrollment to one-year follow-up was increased in the FT1DM group (Δchange = 0.40 [−0.28, 0.80]%) but decreased in the TT1DM group (Δchange = −0.70 [−3.30, 0.55]%, *P*_Δchange_ = 0.286). The WHR in the FT1DM group was significantly higher than that in the TT1DM group both at enrollment and after one-year follow-up. The increase in BMI from enrollment to one-year follow-up was only observed in the TT1DM group. The ln GDR in the FT1DM group at enrollment was significantly higher than that in the TT1DM group (*P* < 0.001), and after one-year follow-up, it was similar between the two groups (*P* = 0.156). Significant decrease of insulin dosage from enrollment to one-year follow-up was also observed in the TT1DM group (*P* = 0.024).

A significant improvement of HDL-C level was observed in the FT1DM group from enrollment to one-year follow-up. The change of HDL-C (ΔHDL‐C) was greater in the FT1DM group than in the TT1DM group (*P* = 0.042). Other indices of lipid profiles including LDL-C, TG, and TC were similar between the two groups both at enrollment and after one year.

At enrollment, fasting and postprandial C-peptide levels in the FT1DM group were significantly lower than those in the TT1DM group (*P* < 0.001, *P* < 0.001). And after one-year follow-up, the low C-peptide level in FT1DM remained unchanged from the enrollment. There was also no significant change on C-peptide level observed in the TT1DM group. Compared with the TT1DM group, the percentage of the positive diabetic antibodies was significantly lower in the FT1DM group with 7.5% in GADA, 3.8% in IA-2A, and none in Zn8TA. The titers of diabetic antibodies were also lower in the FT1DM group especially GADA (0.010 [0.003, 0.020] vs. 0.089 [0.009, 0.681], *P* < 0.001).

## 4. Discussion

Fulminant type 1 diabetes was first described more than twenty years ago [4]; most of the patients with FT1DM have been reported in Asian while a few cases have been reported in the Caucasian population [2, 10, 13, 14]. Using a large multicentre cohort of GTT, the prevalence of FT1DM diagnosed in our study was 3.04%, which was lower than that in Japan [1]. FT1DM in our study was predominately observed in adults, which was similar with the previous study [13]. Previous studies suggested that the onset of FT1DM was associated with pregnancy due to the high rate of onset occurred during pregnancy [1]. In our study, 8 pregnant patients developed diabetes during the second and the third semester and there were 7 of them encountering the fetal demise. Compared with nonpregnant FT1DM patients, pregnancy-associated FT1DM patients were more clinically severe at the onset with more severe acidosis and had higher amylase level and increased incidence of vomiting and infection [15, 16]. Therefore, it is crucial for physicians and obstetricians to pay more attention to early presence and instant treatment for hyperglycaemic symptoms in avoiding abrupt deterioration and poor prognosis.

Symptoms related to hyperglycaemia and ketoacidosis were obviously observed at the onset of FT1DM in our studies. Hypoglycaemia was also reported in some published literatures just before the onset of FT1DM [4]. This is probably because beta-cell destruction is so rapid that insulin in the destroyed beta-cells may enter the blood stream within a short period of time and then resulted in hyperglycaemia without sufficient regulations of more insulin. In our study, the C-peptide level was significantly lower in FT1DM patients at the onset than in TT1DM patients. And after one-year follow-up, the beta-cell function of FT1DM remained as low as that at enrollment. Similar phenomenon was also observed in previous studies. Thus, we infer that there might be no “honeymoon” period and recovery of beta-cell function after the onset of this disease [1, 6, 7]. The absolute beta-cell dysfunction might contribute to the unstable blood glucose even with insulin treatment [17]. In our study, the median HbA1c values were particularly lower in the FT1DM group at enrollment than in the TT1DM group because the duration of the disease was too short. After one-year follow-up, HbA1c level was similar in both groups with the elevation from enrollment in the FT1DM group and the decrease in the TT1DM group. In a following study conducted every 3 months, Imagawa et al. also showed that the HbA1c level between FT1DM and TT1DM was already similar at the first 3 months after the onset [1]. Based on these findings, the beta-cell function seems to be irreversible once destructed and the glycaemic control in FT1DM might be no worse than that in TT1DM with the management of follow-up.

Quantitative lipid abnormalities were observed in patients with type 1 diabetes especially the poorer glycaemic control ones, which may be associated with the development of late diabetic complications [18]. However, few studies focused on the lipid characteristics of FT1DM. In our study, HDL-C level was similar between the FT1DM and TT1DM groups at enrollment. After one-year follow-up, the HDL-C levels in both groups were increased, especially in the FT1DM group. This is probably because the peripheral hyperinsulinemia after insulin treatment activates lecithin-cholesterol acyl transferase and hepatic lipase activities [19]. The Framingham Study showed that HDL-C was a protective factor against cardiovascular disease (CVD) [20]. However, whether the increases in HDL-C level had an inverse association with CVD in patients with T1DM remained controversial. Chiesa et al. recently reported that increased levels of HDL-C may be detrimental to endothelial function when accompanied by renal dysfunction and chronic inflammation [21]. As for the F1TDM, both the viral infection and autoimmunity contributed to the pathogenesis. Whether the contribution for these two pathways affected the HDL-C function and CVD is still unknown.

Insulin resistance (IR) was considered a risk factor for coronary artery disease in adult patients with T1DM [22]. The golden criteria to evaluate IR is glucose disposal rate (GDR) derived by a euglycaemic-hyperinsulinemic clamp, but it is too complicated and time-consuming to be applied. Thus, an eGDR formula was used to assess IR in our study. The ln GDR in FT1DM was significantly higher than that in TT1DM at enrollment. After one-year follow-up, there seemed to be a slight decrease in ln GDR in FT1DM. The deterioration of glycaemic control might contribute mostly to the decrease in ln GDR, which reflected the aggravation of IR. The tendency of higher ln GDR in the FT1DM group should not be neglected because it might contribute to the higher risk of coronary disease.

In our study, a few FT1DM patients were found to have islet antibodies present but with low titers: 4 patients were positive for GADA and 2 patients were found positive for IA-2A while Zn8TA were found all negative. In a nationwide survey of FT1DM in Japan, positive GADA was also detected in some cases but the titer was low and positivity was transient [1]. In a collaborative clinical case investigation in China [3], GADA, IA-2A, and Zn8TA were found positive with 24.5%, 6.1%, and 17.4%, respectively, which were seemly higher than those of the Japanese [1, 3]. Kotani et al. reported that 9 of 13 (69.2%) GAD-reactive Th1 cells and 3 of 12 (25%) insulin-B9-23-reactive Th1 cells were identified in FT1DM by the ELISPOT assay [23]. Wang et al. found that GAD-stimulated interferon-*γ* (IFN-*γ*) and both insulin- and C-peptide-stimulated IFN-*γ* spots were detected in some cases in the Chinese FT1DM population [24]. These results suggested that autoimmune responses might contribute, at least in part, to the development of FT1DM. Based on the findings, the pathophysiology of beta-cell destruction in FT1DM patients remained unclear. Human leukocyte antigen (HLA) typing viral infections and T-cell autoimmunity might be involved [23, 25, 26]. There was also a tentative hypothesis of beta-cell destruction proposed by Imagawa and Hanafusa [5] indicating that virus and its self-replication of the infected cells are the first way to beta-cell death; then, the subsequent innate immune response activated by virus and destruction of beta-cells by T cells would be the second and the third pathway, respectively. Further detailed studies would be necessary to clarify the mechanism of FT1DM pathogenesis.

Several limitations of this study should be addressed. First, for a more accurate comparison, we analyzed the centralised islet antibody value tested at enrollment instead of the reported ones at the onset. However, with the diabetes developed, there might be a negative conversion occurring in some patients, leading to a nonauthentic positive rate or titer. Second, it was accepted that there was a close relationship between pregnancy and FT1DM whereas the number of pregnant patients in our study was insufficient for a powerful analysis of their metabolic characteristics. Lastly, the one-year follow-up duration is relatively short and studies with longer follow-up duration are needed to provide more information about progression of the disease.

## 5. Conclusions

In conclusion, patients with FT1DM in China were not uncommon. With more severe metabolic derangement and deficiency of beta-cell function in FT1DM at the onset, it is required to pay more attention to instant treatment for these patients especially those during pregnancy. After one-year follow-up, glycaemic and metabolic control in FT1DM was not worse than that in TT1DM. Further discussion on the lipid treatment and prevention of complications is required to facilitate more effective management and treatment in the future.

## Figures and Tables

**Table 1:** Clinical characteristics at the onset between FT1DM and TT1DM.

Characteristics	FT1DM (*n* = 53)	TT1DM (*n* = 212)	*P*
Age at onset (years)	30.89 ± 13.08	22.17 ± 12.66	0.445
Age at onset ≥ 18 years (%)	47 (88.7)	179 (84.4)	0.273
Gender (F/M)	31/22	123/89	0.469
BMI (kg/m^2^)	20.92 ± 3.70	19.50 ± 3.97	0.015
Duration of symptoms at onset (days)	2 (1, 7)	30 (10, 60)	<0.001
Onset with DKA (%)	47 (88.7)	85 (40.1)	<0.001
Blood gas analysis			
Arterial pH	7.16 (7.03, 7.27)	7.34 (7.15, 7.39)	<0.001
HCO3^−^ (mmol/l)	7.00 (3.78, 12.85)	15.00 (7.16, 22.65)	0.001
Blood chemistry			
Blood glucose (mmol/l)	34.09 ± 12.35	24.98 ± 9.64	<0.001
HbA1c (%)	6.42 ± 0.72	12.13 ± 3.40	<0.001
*β*-Hydroxybutyric acid (mmol/l)	3.25 (0.96, 6.53)	2.30 (0.60, 4.60)	0.075
K^+^ (mmol/l)	4.80 (3.80, 5.85)	4.09 (3.68, 4.64)	0.002
Na^+^ (mmol/l)	133.0 (129.1, 137.5)	136.1 (132.0, 140.0)	0.046
LDL-C (mmol/l)	2.20 (1.55, 3.01)	2.75 (1.96, 3.49)	0.006
HDL-C (mmol/l)	1.12 (0.79, 1.50)	1.19 (1.00, 1.40)	0.404
TG (mmol/l)	1.17 (0.80, 2.43)	1.20 (0.81, 1.96)	0.799
TC (mmol/l)	4.35 (3.16, 5.80)	4.70 (4.01, 5.87)	0.140
CBC			
WBC (10*E*^9^/l)	20.91 ± 10.34	10.45 ± 7.25	<0.001
Neutrophil (10*E*^9^/l)	16.63 ± 9.20	7.46 ± 6.87	<0.001
Accompanied symptoms			
Pancreatitis (%)	9 (17.0)	3 (1.4)	<0.001
Conscious (%)	19 (35.8)	30 (14.2)	<0.001
Infection (%)	15 (28.2)	33 (15.6)	<0.001
Endocrine examination			
FCP (nmol/L)	0.01 (0.00, 0.03)	0.09 (0.04, 0.23)	<0.001
PCP (nmol/L)	0.02 (0.01, 0.05)	0.20 (0.09, 0.54)	<0.001
Autoantibodies^†^			
GADA positive (%)	4 (7.5)	77 (36.3)	<0.001
GADA titer	0.010 (0.003, 0.020)	0.089 (0.009, 0.681)	<0.001
Zn8TA positive (%)	0 (0.0)	22 (10.4)	0.024
Zn8TA titer	0.005 (-0.007, 0.012)	0.009 (-0.004, 0.022)	0.056
IA-2A positive (%)	2 (3.8)	36 (17.0)	0.042
IA-2A titer	-0.003 (-0.010, 0.002)	0.000 (-0.004, 0.025)	0.016

Data are presented as mean ± SD or median (interquartile range) for continuous variables and % (*n*) for categorical variables. ^†^Antibodies presented here were tested at enrollment. Abbreviation: FT1DM: fulminant type 1 diabetes mellitus; TT1DM: typical type 1 diabetes mellitus; F: female; M: male; BMI: body mass index; HbA1c: glycated haemoglobin; pH: potential of hydrogen; LDL-C: low-density lipoprotein cholesterol; HDL-C: high-density lipoprotein cholesterol; TG: triglyceride; TC: total cholesterol; ALT: alanine aminotransferase; AST: alanine aminotransferase; WBC: white blood cell; DKA: diabetic ketoacidosis; FCP: fasting C-peptide; PCP: postprandial 2-hour C-peptide; GADA: glutamic acid decarboxylase antibody; IA-2A: insulinoma-associated protein 2/islet cell antigen 512 antibody; ZnT8A: zinc transporter 8 antibody.

**Table 2:** Clinical characteristics during one-year follow-up between FT1DM and TT1DM.

	FT1DM (*n* = 53)		*P*	TT1DM (*n* = 212)		*P*
	At enrollment	1-year follow-up		At enrollment	1-year follow-up	
Diabetic duration (years)	0.03 (0.00, 0.81)	/	/	0.07 (0.00, 1.11)	/	/
HbA1c (%)	7.21 ± 1.56^∗^	7.67 ± 1.69	0.034	10.06 ± 3.23	8.40 ± 2.38	<0.001
Dosage of insulin (IU/kg/d)	0.72 ± 0.25	0.68 ± 0.17	0.351	0.71 ± 0.32	0.67 ± 0.30	0.024
WHR	0.83 ± 0.07^∗^	0.82 ± 0.06^∗^	0.388	0.85 ± 0.08	0.85 ± 0.08	0.530
ln GDR^†^	2.08 ± 0.24^∗^	2.01 ± 0.29	0.089	1.70 ± 0.42	1.91 ± 0.36	<0.001
BMI (kg/m^2^)	20.43 ± 3.15	20.18 ± 2.04	0.726	20.07 ± 3.14	20.58 ± 3.06	<0.001
LDL-C (mmol/l)	2.39 (1.70, 3.10)	2.34 (1.98, 3.00)	0.675	2.70 (1.97, 3.46)	2.63 (2.06, 3.14)	0.408
HDL-C (mmol/l)	1.45 ± 0.47	1.85 ± 0.54	0.019	1.46 ± 0.49	1.67 ± 0.43	0.162
TG (mmol/l)	0.82 (0.61, 1.07)	0.55 (0.50, 0.72)	0.875	0.93 (0.66, 1.38)	0.77 (0.59, 1.17)	0.507
TC (mmol/l)	4.59 ± 1.30	4.67 ± 1.00	0.600	4.86 ± 1.27	4.83 ± 0.94	0.149
FCP (nmol/l)	0.06 (0.03, 0.13)^∗^	0.05 (0.02, 0.16)^∗^	0.637	0.21 (0.08, 0.40)	0.24 (0.05, 0.58)	0.955
PCP (nmol/l)	0.06 (0.03, 0.14)^∗^	0.06 (0.03, 0.14)^∗^	0.437	0.30 (0.13, 0.72)	0.32 (0.11, 0.84)	0.145

Dara were presented as mean ± standard deviation or median (interquartile range) for continuous variables and % for categorical variables. ^†^ln GDR was calculated among adult patients in the respective group. ^∗^*P* < 0.05, comparison of data between the FT1DM and TT1DM groups. Abbreviations: FT1DM: fulminant type 1 diabetes; TT1DM: typical type 1 diabetes; WHR: waist/height ratio; GDR: glucose disposal rate; BMI: body mass index; LDL-C: low-density lipoprotein cholesterol; HDL-C: high-density lipoprotein; TG: triglyceride; TC: total cholesterol; FCP: fasting C-peptide; PCP: postprandial C-peptide.

## Data Availability

The data used to support the findings of this study are available from the corresponding author upon request.
